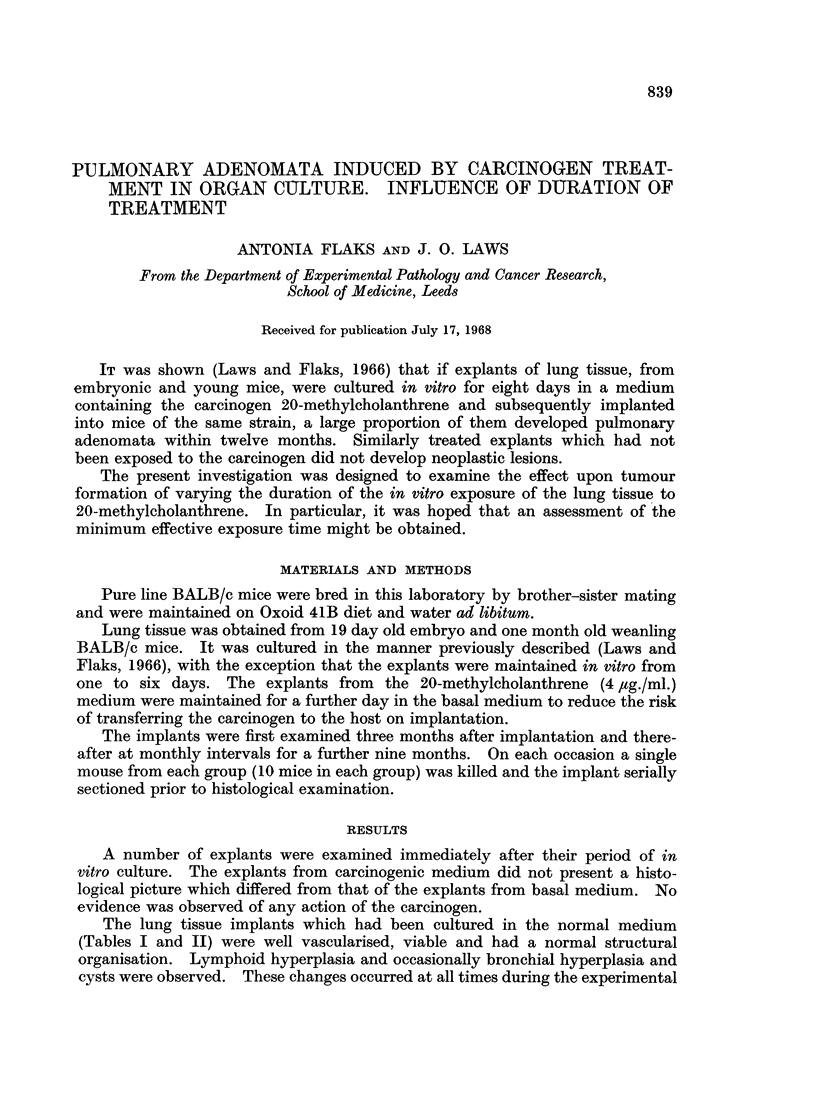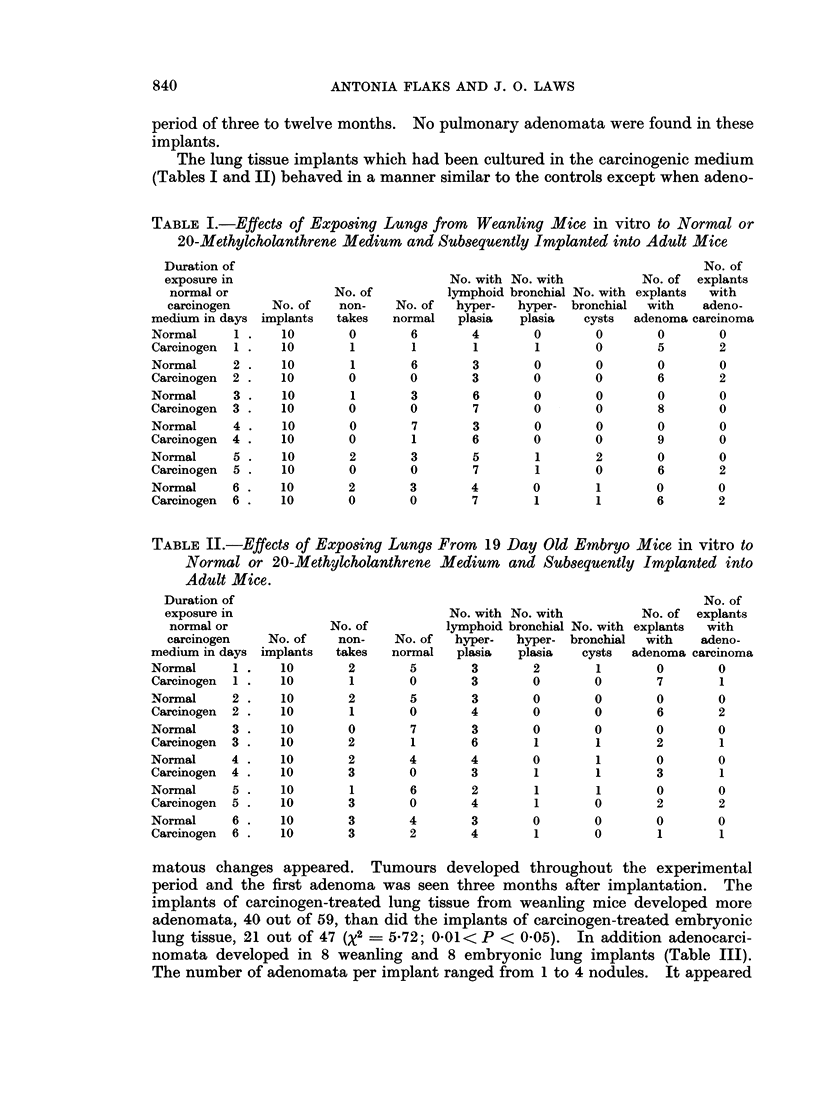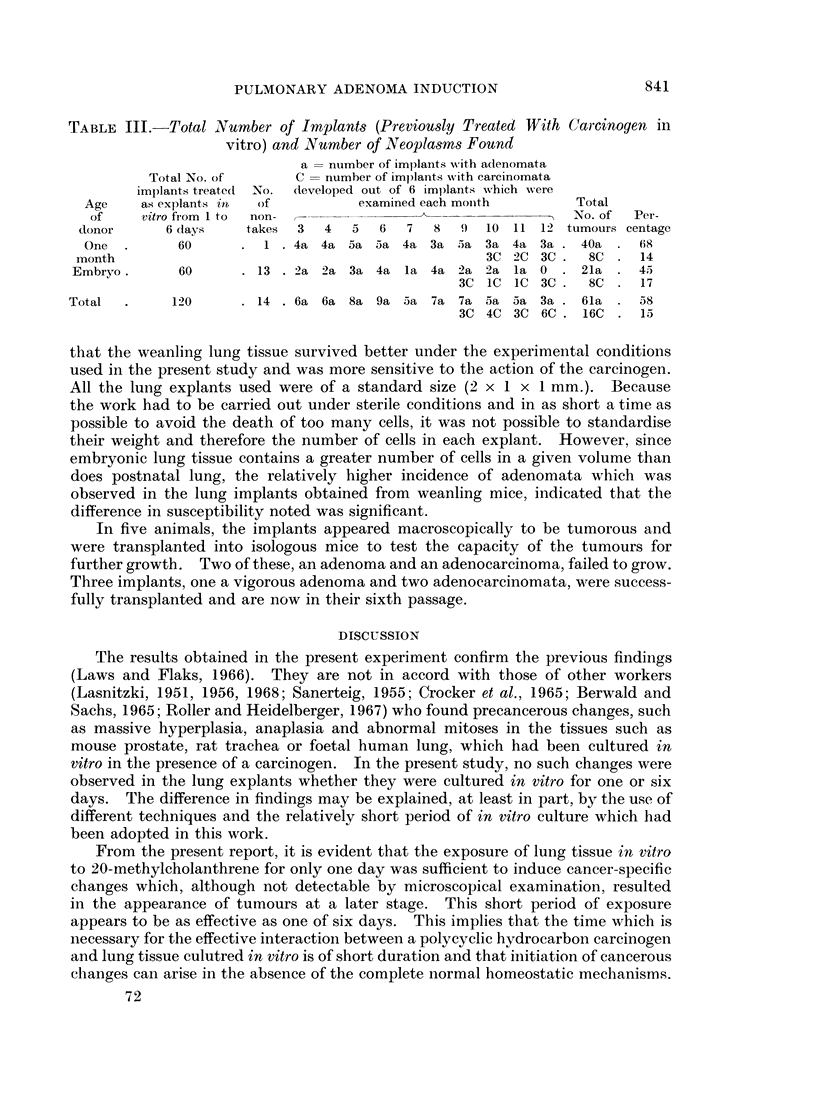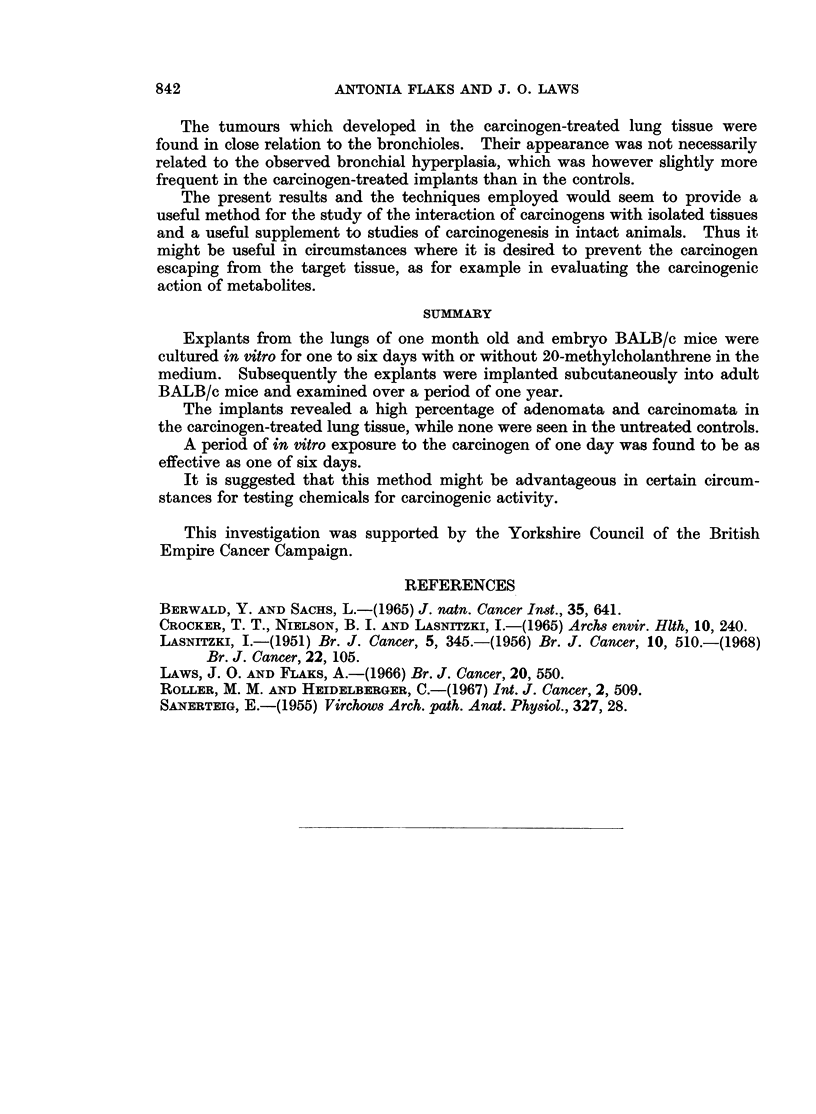# Pulmonary Adenomata Induced by Carcinogen Treatment in Organ Culture. Influence of Duration of Treatment

**DOI:** 10.1038/bjc.1968.98

**Published:** 1968-12

**Authors:** Antonia Flaks, J. O. Laws


### PULMONARY ADENOMATA INDUCED BY CARCINOGEN TREATMENT IN ORGAN CULTURE. INFLUENCE OF DURATION OF TREATMENT

ANTONIA FLAKS AND J. O. LAWS

*From the Department of Experimental Pathology and Cancer Research, School of Medicine, Leeds*

Received for publication July 17, 1968

It was shown (Laws and Flaks, 1966) that if explants of lung tissue, from embryonic and young mice, were cultured *in vitro* for eight days in a medium containing the carcinogen 20-methylcholanthrene and subsequently implanted into mice of the same strain, a large proportion of them developed pulmonary adenomata within twelve months. Similarly treated explants which had not been exposed to the carcinogen did not develop neoplastic lesions.

The present investigation was designed to examine the effect upon tumour formation of varying the duration of the *in vitro* exposure of the lung tissue to 20-methylcholanthrene. In particular, it was hoped that an assessment of the minimum effective exposure time might be obtained.

### MATERIALS AND METHODS

Pure line BALB/c mice were bred in this laboratory by brother–sister mating and were maintained on Oxoid 41B diet and water *ad libitum*.

Lung tissue was obtained from 19 day old embryo and one month old weanling BALB/c mice. It was cultured in the manner previously described (Laws and Flaks, 1966), with the exception that the explants were maintained *in vitro* from one to six days. The explants from the 20-methylcholanthrene (4 $\mu$g./ml.) medium were maintained for a further day in the basal medium to reduce the risk of transferring the carcinogen to the host on implantation.

The implants were first examined three months after implantation and thereafter at monthly intervals for a further nine months. On each occasion a single mouse from each group (10 mice in each group) was killed and the implant serially sectioned prior to histological examination.

### RESULTS

A number of explants were examined immediately after their period of *in vitro* culture. The explants from carcinogenic medium did not present a histological picture which differed from that of the explants from basal medium. No evidence was observed of any action of the carcinogen.

The lung tissue implants which had been cultured in the normal medium (Tables I and II) were well vascularised, viable and had a normal structural organisation. Lymphoid hyperplasia and occasionally bronchial hyperplasia and cysts were observed. These changes occurred at all times during the experimental period of three to twelve months. No pulmonary adenomata were found in these implants.

The lung tissue implants which had been cultured in the carcinogenic medium (Tables I and II) behaved in a manner similar to the controls except when adenomatous changes appeared. Tumours developed throughout the experimental period and the first adenoma was seen three months after implantation. The implants of carcinogen-treated lung tissue from weanling mice developed more adenomata, 40 out of 59, than did the implants of carcinogen-treated embryonic lung tissue, 21 out of 47 ($\chi^2 = 5{\cdot}72$; $0{\cdot}01 < P < 0{\cdot}05$). In addition adenocarcinomata developed in 8 weanling and 8 embryonic lung implants (Table III). The number of adenomata per implant ranged from 1 to 4 nodules. It appeared

TABLE I.—*Effects of Exposing Lungs from Weanling Mice* in vitro *to Normal or 20-Methylcholanthrene Medium and Subsequently Implanted into Adult Mice*

| Duration of exposure in normal or carcinogen medium in days | No. of implants | No. of non-takes | No. of normal | No. with lymphoid hyper-plasia | No. with bronchial hyper-plasia | No. with bronchial cysts | No. of explants with adenoma | No. of explants with adeno-carcinoma |
|---|---|---|---|---|---|---|---|---|
| Normal 1 . | 10 | 0 | 6 | 4 | 0 | 0 | 0 | 0 |
| Carcinogen 1 . | 10 | 1 | 1 | 1 | 1 | 0 | 5 | 2 |
| Normal 2 . | 10 | 1 | 6 | 3 | 0 | 0 | 0 | 0 |
| Carcinogen 2 . | 10 | 0 | 0 | 3 | 0 | 0 | 6 | 2 |
| Normal 3 . | 10 | 1 | 3 | 6 | 0 | 0 | 0 | 0 |
| Carcinogen 3 . | 10 | 0 | 0 | 7 | 0 | 0 | 8 | 0 |
| Normal 4 . | 10 | 0 | 7 | 3 | 0 | 0 | 0 | 0 |
| Carcinogen 4 . | 10 | 0 | 1 | 6 | 0 | 0 | 9 | 0 |
| Normal 5 . | 10 | 2 | 3 | 5 | 1 | 2 | 0 | 0 |
| Carcinogen 5 . | 10 | 0 | 0 | 7 | 1 | 0 | 6 | 2 |
| Normal 6 . | 10 | 2 | 3 | 4 | 0 | 1 | 0 | 0 |
| Carcinogen 6 . | 10 | 0 | 0 | 7 | 1 | 1 | 6 | 2 |

TABLE II.—*Effects of Exposing Lungs From* 19 *Day Old Embryo Mice* in vitro *to Normal or 20-Methylcholanthrene Medium and Subsequently Implanted into Adult Mice.*

| Duration of exposure in normal or carcinogen medium in days | No. of implants | No. of non-takes | No. of normal | No. with lymphoid hyper-plasia | No. with bronchial hyper-plasia | No. with bronchial cysts | No. of explants with adenoma | No. of explants with adeno-carcinoma |
|---|---|---|---|---|---|---|---|---|
| Normal 1 . | 10 | 2 | 5 | 3 | 2 | 1 | 0 | 0 |
| Carcinogen 1 . | 10 | 1 | 0 | 3 | 0 | 0 | 7 | 1 |
| Normal 2 . | 10 | 2 | 5 | 3 | 0 | 0 | 0 | 0 |
| Carcinogen 2 . | 10 | 1 | 0 | 4 | 0 | 0 | 6 | 2 |
| Normal 3 . | 10 | 0 | 7 | 3 | 0 | 0 | 0 | 0 |
| Carcinogen 3 . | 10 | 2 | 1 | 6 | 1 | 1 | 2 | 1 |
| Normal 4 . | 10 | 2 | 4 | 4 | 0 | 1 | 0 | 0 |
| Carcinogen 4 . | 10 | 3 | 0 | 3 | 1 | 1 | 3 | 1 |
| Normal 5 . | 10 | 1 | 6 | 2 | 1 | 1 | 0 | 0 |
| Carcinogen 5 . | 10 | 3 | 0 | 4 | 1 | 0 | 2 | 2 |
| Normal 6 . | 10 | 3 | 4 | 3 | 0 | 0 | 0 | 0 |
| Carcinogen 6 . | 10 | 3 | 2 | 4 | 1 | 0 | 1 | 1 |

TABLE III.—*Total Number of Implants (Previously Treated With Carcinogen* in vitro) *and Number of Neoplasms Found*

| Age of donor | Total No. of implants treated as explants *in vitro* from 1 to 6 days | No. of non-takes | a = number of implants with adenomata C = number of implants with carcinomata developed out of 6 implants which were examined each month: 3 | 4 | 5 | 6 | 7 | 8 | 9 | 10 | 11 | 12 | Total No. of tumours | Percentage |
|---|---|---|---|---|---|---|---|---|---|---|---|---|---|---|
| One month | 60 | 1 | 4a | 4a | 5a | 5a | 4a | 3a | 5a | 3a | 4a | 3a | 40a | 68 |
| | | | | | | | | | | 3C | 2C | 3C | 8C | 14 |
| Embryo | 60 | 13 | 2a | 2a | 3a | 4a | 1a | 4a | 2a | 2a | 1a | 0 | 21a | 45 |
| | | | | | | | | | 3C | 1C | 1C | 3C | 8C | 17 |
| Total | 120 | 14 | 6a | 6a | 8a | 9a | 5a | 7a | 7a | 5a | 5a | 3a | 61a | 58 |
| | | | | | | | | | 3C | 4C | 3C | 6C | 16C | 15 |

that the weanling lung tissue survived better under the experimental conditions used in the present study and was more sensitive to the action of the carcinogen. All the lung explants used were of a standard size (2 × 1 × 1 mm.). Because the work had to be carried out under sterile conditions and in as short a time as possible to avoid the death of too many cells, it was not possible to standardise their weight and therefore the number of cells in each explant. However, since embryonic lung tissue contains a greater number of cells in a given volume than does postnatal lung, the relatively higher incidence of adenomata which was observed in the lung implants obtained from weanling mice, indicated that the difference in susceptibility noted was significant.

In five animals, the implants appeared macroscopically to be tumorous and were transplanted into isologous mice to test the capacity of the tumours for further growth. Two of these, an adenoma and an adenocarcinoma, failed to grow. Three implants, one a vigorous adenoma and two adenocarcinomata, were successfully transplanted and are now in their sixth passage.

### DISCUSSION

The results obtained in the present experiment confirm the previous findings (Laws and Flaks, 1966). They are not in accord with those of other workers (Lasnitzki, 1951, 1956, 1968; Sanerteig, 1955; Crocker *et al.*, 1965; Berwald and Sachs, 1965; Roller and Heidelberger, 1967) who found precancerous changes, such as massive hyperplasia, anaplasia and abnormal mitoses in the tissues such as mouse prostate, rat trachea or foetal human lung, which had been cultured *in vitro* in the presence of a carcinogen. In the present study, no such changes were observed in the lung explants whether they were cultured *in vitro* for one or six days. The difference in findings may be explained, at least in part, by the use of different techniques and the relatively short period of *in vitro* culture which had been adopted in this work.

From the present report, it is evident that the exposure of lung tissue *in vitro* to 20-methylcholanthrene for only one day was sufficient to induce cancer-specific changes which, although not detectable by microscopical examination, resulted in the appearance of tumours at a later stage. This short period of exposure appears to be as effective as one of six days. This implies that the time which is necessary for the effective interaction between a polycyclic hydrocarbon carcinogen and lung tissue culutred *in vitro* is of short duration and that initiation of cancerous changes can arise in the absence of the complete normal homeostatic mechanisms.

The tumours which developed in the carcinogen-treated lung tissue were found in close relation to the bronchioles. Their appearance was not necessarily related to the observed bronchial hyperplasia, which was however slightly more frequent in the carcinogen-treated implants than in the controls.

The present results and the techniques employed would seem to provide a useful method for the study of the interaction of carcinogens with isolated tissues and a useful supplement to studies of carcinogenesis in intact animals. Thus it might be useful in circumstances where it is desired to prevent the carcinogen escaping from the target tissue, as for example in evaluating the carcinogenic action of metabolites.

### SUMMARY

Explants from the lungs of one month old and embryo BALB/c mice were cultured *in vitro* for one to six days with or without 20-methylcholanthrene in the medium. Subsequently the explants were implanted subcutaneously into adult BALB/c mice and examined over a period of one year.

The implants revealed a high percentage of adenomata and carcinomata in the carcinogen-treated lung tissue, while none were seen in the untreated controls.

A period of *in vitro* exposure to the carcinogen of one day was found to be as effective as one of six days.

It is suggested that this method might be advantageous in certain circumstances for testing chemicals for carcinogenic activity.

This investigation was supported by the Yorkshire Council of the British Empire Cancer Campaign.

### REFERENCES

Berwald, Y. and Sachs, L.—(1965) *J. natn. Cancer Inst.*, **35**, 641.
Crocker, T. T., Nielson, B. I. and Lasnitzki, I.—(1965) *Archs envir. Hlth*, **10**, 240.
Lasnitzki, I.—(1951) *Br. J. Cancer*, **5**, 345.—(1956) *Br. J. Cancer*, **10**, 510.—(1968) *Br. J. Cancer*, **22**, 105.
Laws, J. O. and Flaks, A.—(1966) *Br. J. Cancer*, **20**, 550.
Roller, M. M. and Heidelberger, C.—(1967) *Int. J. Cancer*, **2**, 509.
Sanerteig, E.—(1955) *Virchows Arch. path. Anat. Physiol.*, **327**, 28.